# Reinforcement drives within- not between-trial motor adaptation

**DOI:** 10.1038/s41598-026-45293-8

**Published:** 2026-04-04

**Authors:** Finn M. Lehnberg, Theresa Paul, Valerie M. Wiemer, Scott T. Grafton, Gereon R. Fink, Lukas J. Volz

**Affiliations:** 1 Medical Faculty, University of Cologne, and Department of Neurology, University Hospital Cologne, 50937 Cologne, Germany; 2 Institute of Neuroscience and Medicine, Cognitive Neuroscience (INM-3), Research Centre Juelich, 52428 Juelich, Germany; 3 Department of Psychological & Brain Sciences, University of California, Santa Barbara, CA 93106 United States of America

**Keywords:** Motor planning, Visuomotor rotation, Motor variability, Sensory prediction error, Online correction, Neuroscience, Psychology, Psychology

## Abstract

While reinforcement has repeatedly been shown to impact motor adaptation, previous inconsistent findings hinder our mechanistic understanding. To conciliate contradictory views, we used two distinct motor tasks to investigate whether reinforcement differentially modulates two core motor learning processes: *between-trial* learning resulting from motor planning and *within-trial* learning supported by the online correction of ongoing movements. We observed successful adaptation, retention and highly similar temporal profiles of adaptation for both tasks, in line with the notion that motor adaptation of upper limb movements is likely to result from updates in both motor planning and online correction. Reinforcement enhanced performance in the task allowing for *within-trial* adaptation. Conversely, higher degrees of early motor variability were associated with faster *between-trial* but not *within-trial* adaptation. These results highlight a dissociation of factors affecting motor adaptation: Reinforcement seems to primarily impact *within-trial* learning linked to the online correction of ongoing movements. In contrast, early motor variability might benefit *between-trial* learning by informing motor planning potentially via an exploration of the motor output space. Our results help explain previous inconsistent findings and add to our theoretical understanding by demonstrating that the effects of reinforcement and motor variability may depend on whether adaptation is driven by *within*- or *between-trial* learning.

**Supplementary Information:**

The online version contains supplementary material available at 10.1038/s41598-026-45293-8.

## Introduction

Motor control arises from various processes that contribute to the planning, initiation and execution of movements^1,2^. Allowing us to interact with our dynamic environments successfully, motor adaptation enables the flexible updating of motor control policies in an error-based fashion^3,4^. In particular, sensory prediction errors (SPEs), i.e., the mismatch between the predicted and actual sensory consequences of a movement, are central to successful motor adaptation^1,5^. When experimentally induced via visuomotor rotation, SPEs can inform immediate online correction to adjust ongoing movements, a process that enables *within-trial* learning during adaptation^6,7^. However, adaptation has also been shown to occur in the absence of continuous visual feedback^8^, highlighting that modifying motor planning, e.g., via planning different follow-through movements, can facilitate motor adaptation^9^. Here, SPEs and target errors from previous attempts may inform updates to the motor plan for subsequent movements, resulting in *between-trial* learning.

Motor adaptation has repeatedly been shown to be sensitive to reinforcement, but the reported effects are inconsistent^10–16^. In these studies, reinforcement was typically implemented via monetary gains or losses, often signalled by points or auditory feedback, contingent on performance outcomes. A potential explanation for the inconsistency of these effects may derive from the task-specific balance of *within-trial*^14^ and *between-trial*^15^ feedback and learning. For example, punishment showed opposite effects in a task providing error-based *within-trial* feedback^14^ compared to a task without it^15^. Even though there is evidence showing that reinforcement modulates properties and neural correlates of both movement planning and online correction^5,17–19^, prior reinforcement adaptation studies typically employed tasks in which subjects retained continuous movement control allowing for online corrections of ongoing movements, even when instructed to make “shooting” movements or to perform movements without visual feedback^12,14–16^.

An additional explanation for the varying effects of reinforcement may derive from its differential impact on motor variability. During adaptation, a search of the motor output space may help the motor system to define near-optimal motor control policies to enhance motor learning^20^. In this context, punishment has been reported to increase^21^ or decrease variability^15^, or to have no effect^14^. However, the role of motor variability in adaptation remains debated^22^. A theoretical framework trying to resolve this issue proposed distinct components of early motor variability with beneficial “planning noise” facilitating and “execution noise” hindering motor adaptation^23^. Similarly, motor variability may serve distinct roles in *within-* and *between-trial* learning. While “planning noise” may be informative for *between-trial* learning arising from updated motor plans, “execution noise” may hinder or “dampen” *within-trial* learning resulting from online corrections. Taken together, these rationales lead to the central questions of whether *within-* and *between-trial* learning are distinctly modulated by reinforcement and differentially informed by early motor variability.

To address these questions, we used two visuomotor adaptation joystick tasks to disentangle *within*- and *between-trial* learning during motor adaptation. One task permitted online corrections, thus engaging both *within*- and *between-trial* learning, while the other relied on *between-trial* learning by preventing online corrections. Given that modifications on the level of motor planning, such as planning different follow-through movements or an explicit aiming direction, have been shown to facilitate adaptation^3,9^, we expected successful motor adaptation during both tasks. We hypothesised faster adaptation and better performance when online correction driven by continuous *within-trial* SPEs was available besides *between-trial* learning^24^. Since reinforcement signals, such as appetitive outcomes in rodents or monetary reward in humans, have recently been linked to cerebellar-basal ganglia loops involved in online corrections of ongoing movements^18,19^, we hypothesised reinforcement to primarily modulate *within-trial* learning, with little to no impact on the task relying solely on *between-trial* learning. Building on a theoretical framework suggesting that planning-related variability may act as a driver of trial-to-trial updates, we reasoned that motor variability would differentially inform adaptation in our tasks^23,25^. Specifically, we expected motor variability to primarily inform *between-trial* but not *within-trial* learning.

## Results

**Fig. 1:**
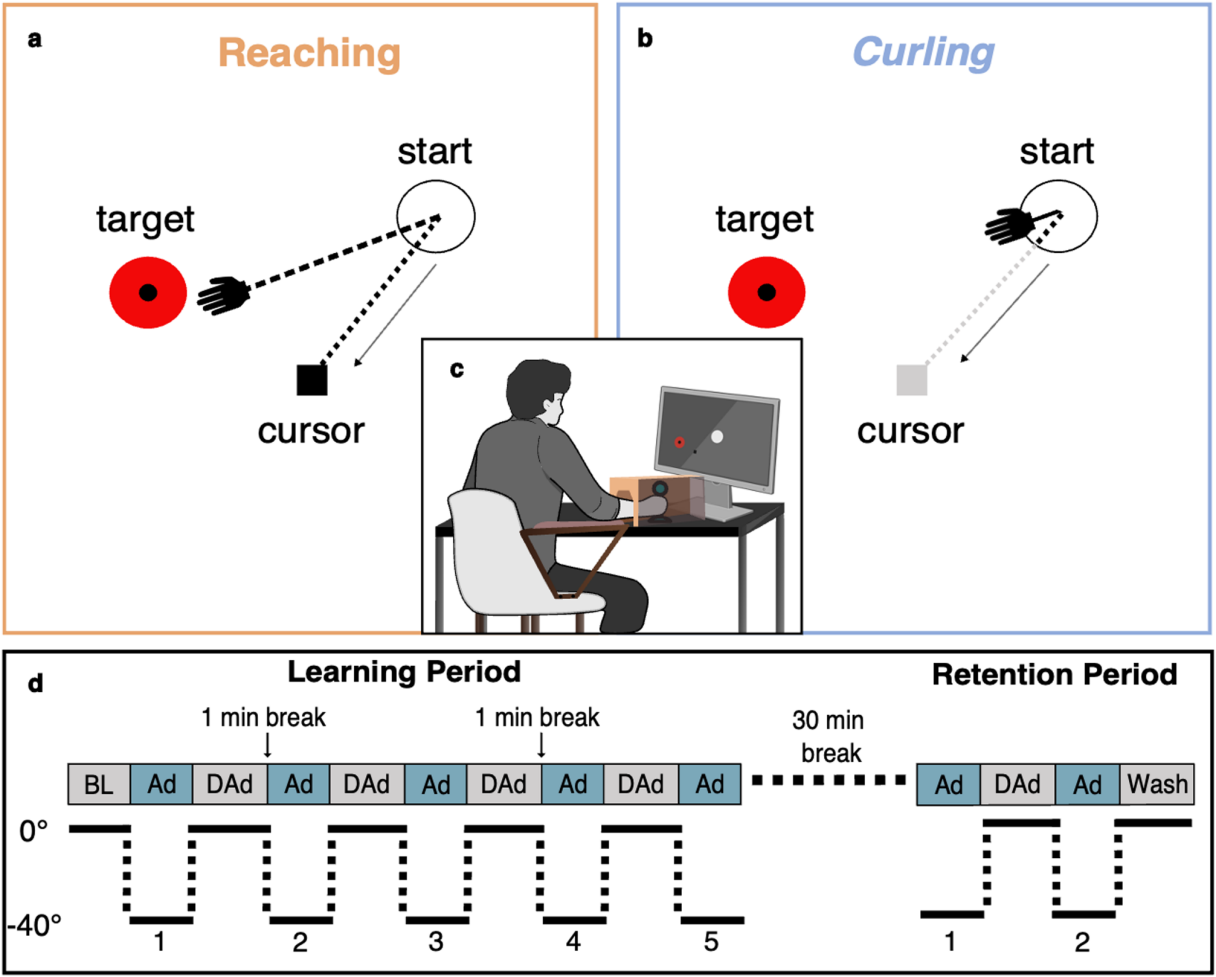
Task setup and schedule. **(a)** In the *Reaching* task, participants performed reaching movements with a joystick guiding the cursor to the target, with online corrections of the movement trajectory being possible throughout the whole trial. **(b)** In the *Curling* task, participants initiated a movement until an 8 mm radius was crossed, representing the “launch phase” of each trial. The cursor then continued moving along the initiated trajectory (grey trajectory), independent of further joystick movements (“autonomous phase”). For exemplary trajectory depiction see Supplementary Fig. S4. **(a)** & **(b)** Depict rotated adaptation (Ad 1–5) trials, where the cursor was rotated 40° counterclockwise to the joystick movement. **(c)** Schematic illustration of the experimental setup, showing a seated subject controlling the joystick. The vision of the hand and arm was occluded to prevent any direct visual feedback on the hand and arm position. **(d)** Task schedule for both tasks. Blocks of 40 trials were performed, including baseline (BL), adaptation (Ad), de-adaptation (DAd) and washout (Wash) trials. In the adaptation trials (depicted in blue), a counterclockwise visuomotor rotation of 40° was introduced. During the *Learning* period, subjects received performance-dependent monetary reward or punishment feedback, or no feedback (neutral condition). The *Retention* period began with a rotated block to capture retention of the updated motor control policy after a 30-minute break. Notably, subjects received no performance-dependent feedback during this period regardless of their respective reinforcement condition.

We used two visuomotor adaptation tasks to disentangle the contributions of *between-trial* versus *within-trial* learning to motor adaptation and their respective modulation by reinforcement. Of note, we used the term *adaptation* when referring to the rapid, error-based adjustment of motor performance in response to the visuomotor perturbation, whereas the more general term *learning* was utilised to describe across-block changes in performance (including savings, retention and washout) and when referring to underlying *within-* and *between-trial* learning mechanisms at a conceptual level. The *Reaching* task allowed for continuous online corrections of movement trajectories or *within-trial* learning. Conversely, during the *Curling* task, the cursor followed a ballistic path based on the very short initial movement direction, not allowing for online correction of the cursor’s trajectory, hence prohibiting *within-trial* learning. Motor adaptation was experimentally induced by a 40° counterclockwise visuomotor rotation of the cursor on the screen. Both tasks followed an identical schedule (Fig. 1d), including a *Learning* and *Retention* period with alternating rotated and unrotated blocks of 40 trials each. In both tasks, performance was quantified by angular errors (model-free) and learning rates extracted from a single-rate state-space model (model-based)^14^. We tested for reinforcement effects in a between-subjects design, with participants receiving either performance-dependent reward or punishment, or neutral feedback (for further details, please see Materials & Methods).

### Successful adaptation in both tasks with highly similar temporal learning profiles

Participants successfully adapted to the visuomotor rotation in both *Reaching* and *Curling*, demonstrating robust adaptation with and without *within-trial* adaptation. Mixed-effects modelling confirmed adaptation across the *Learning* period, as indicated by a significant main effect of block (χ²_(4)_ = 759.84, *p* < 0.001) reflecting systematic error reduction over successive adaptation blocks. There was no significant task x block interaction (χ²_(4)_ = 0.56, *p* = 0.46), suggesting similar degrees of adaptation across blocks for both tasks. Accordingly, mean angular errors per block significantly decreased for both *Reaching* (F_(4,260)_ = 40.77, *p* < 0.001, generalised η^2^ = 0.16) and *Curling* (F_(4,260)_ = 20.29, *p* < 0.001, generalised η^2^ = 0.089; for further details see Supplementary Fig. S1).

Post-hoc tests indeed revealed that subjects did not further improve between consecutive adaptation blocks after the third adaptation block for either task in model-free analyses (all *p* > 0.05, Supplementary Fig. S1 a and b), suggesting that a performance plateau was reached. Moreover, savings were found for both *Reaching* (F_(4,260)_ = 30.7, *p* < 0.001, generalised η^2^ = 0.18) and *Curling* (F_(4,260)_ = 25.08, *p* < 0.001, generalised η^2^ = 0.14) when comparing the first five trials across adaptation blocks.

In line with the model-free results, the model-based analyses yielded significant improvements in learning rates across blocks for both *Reaching* (χ² = 68.85, *p* < 0.001) and *Curling* (χ² = 44.79, *p* < 0.001). Notably, the across-block progression of learning rates and angular errors was highly similar for both tasks, with an initial stagnation of improvement after three adaptation blocks in both model-free and model-based block-wise comparisons (see Supplementary Fig. S1). Hence, motor adaptation followed highly similar temporal profiles for both tasks. Moreover, subjects showed successful motor adaptation in every adaptation block (Adaptation 1–5). A comparison of early performance (first five trials per block) to late performance (last five trials per block) revealed a significant reduction in angular errors in every block for both *Reaching* (all blocks: *p* < 0.001, FDR-corrected) and *Curling* (all blocks: *p* < 0.001, FDR-corrected).

### Online correction enhances adaptation

**Fig. 2:**
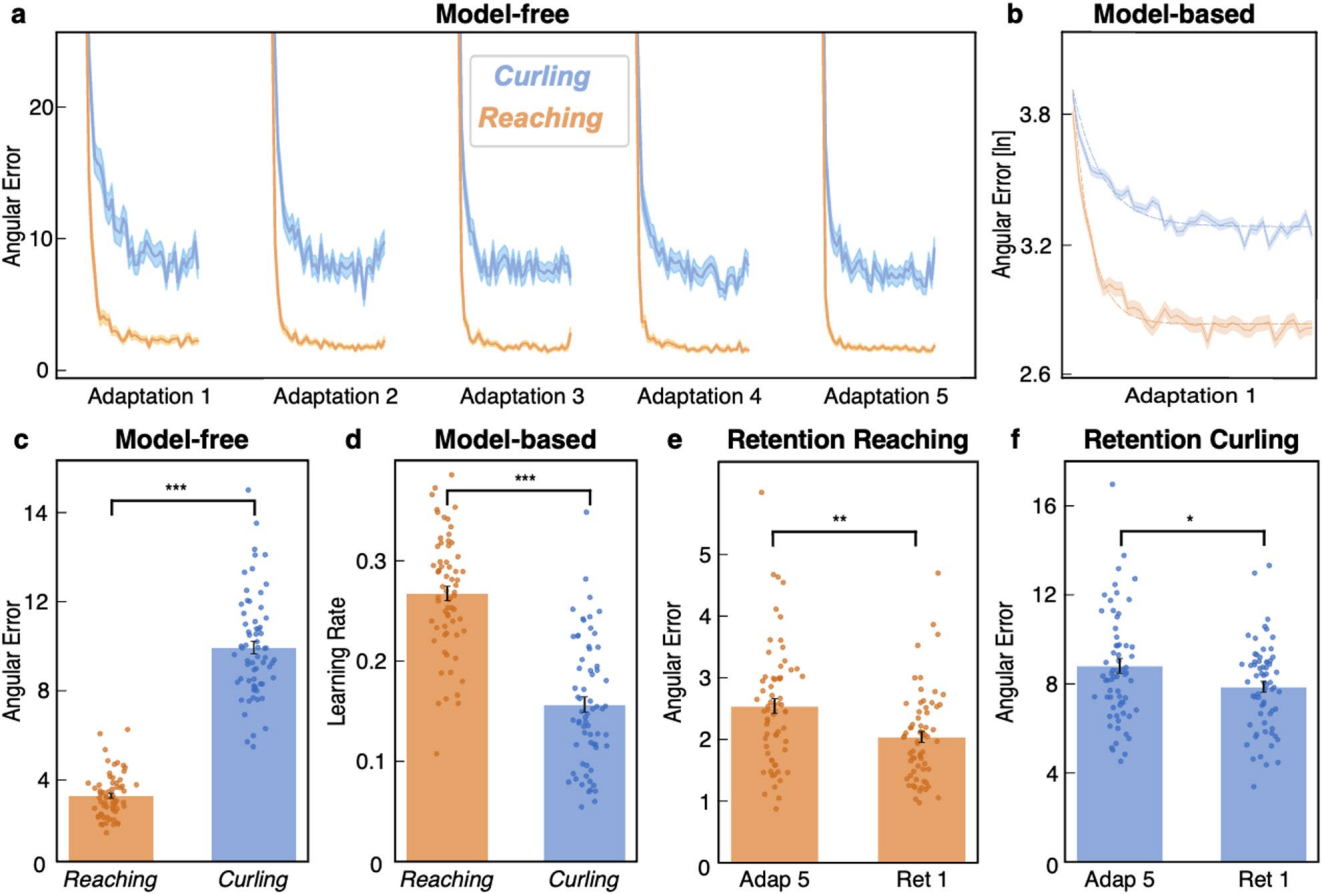
Comparison of *Reaching* and *Curling.* **(a)** Mean angular errors (± SEM) across adaptation blocks of the *Learning* period showed similar temporal adaptation profiles for both *Reaching* (*orange*) and *Curling (blue)*. **(b)** A single-rate model was fitted to the data of both tasks to quantify learning rates (example of model fit shown for Adaptation 1). **(c)** Across all adaptation blocks, angular errors were significantly lower for *Reaching* compared to *Curling*. **(d)** Accordingly, model-based learning rates were significantly higher in *Reaching* compared to *Curling* across all adaptation blocks. Thus, the availability of online correction enabled faster learning. **(e)** Comparing the last adaptation block of the *Learning* period (Adap 5) to the first adaptation block of the *Retention* period (Ret 1) yielded significantly lower angular errors after the break for *Reaching*, indicative of successful retention. **(f)** Successful retention was similarly observed for *Curling*. (* *p* < 0.05, ** *p* < 0.01, and *** *p* < 0.001).

A direct comparison of both tasks via a mixed-effects model revealed that the availability of *within-trial* adaptation conferred a significant performance advantage (main effect of task: χ²_(1)_ = 789.47, *p* < 0.001). Post-hoc paired-samples t-tests were computed and revealed lower angular errors (t_(65)_ = 22.749, *p* < 0.001; Fig. 2c) and higher learning rates (t_(65)_ = 10.94, *p* < 0.001; Fig. 2d) in the *Reaching* compared to the *Curling* task, both across all blocks as well as in each adaptation block separately (*p* < 0.01 for each block). These findings suggest that despite the similarity in temporal characteristics of motor adaptation, the combination of *within-trial* and *between-trial* adaptation during *Reaching* led to a pronounced reduction of angular errors and higher learning rates compared to *between-trial* adaptation alone.

To assess whether superior performance in the *Reaching* task was indeed driven by online correction, we recomputed angular errors in the *Reaching* task at the same 8 mm radius used as the “release point” in *Curling* (see Supplementary Fig. S5). Interestingly, at this matched radial distance, subjects showed significantly higher angular errors in *Reaching* compared to *Curling* (t_(65)_ = 5.93, *p* < 0.001). This indicates that superior performance in *Reaching* compared to *Curling* was driven by online corrections of the ongoing reaching movement reducing the initial angular error reflecting *within-trial* adaptation. Trajectory analyses further corroborated that online corrections were exclusively possible in *Reaching* but not *Curling*. First, *Reaching* trajectories were markedly more curved and showed multiple changes in movement direction (mean 4.86 +/- SD 1.90) compared to *Curling* (mean 0.85, +/- SD 0.40; t_(65)_ = 17.45, *p* < 0.001; see Supplementary Fig. S4b). Additionally, the control time of the cursor during *Reaching* (mean 533.05ms +/- SD 150) was significantly longer than in *Curling* (mean 100.20ms +/- SD 26.25ms; t_(65)_ = 23.90; *p* < 0.001; see Supplementary Fig. S4c). Importantly, the visuomotor feedback latency of 100-150ms needed to adjust ongoing movements^26,27^ renders online corrections during *Curling* highly unlikely.

### Successful retention and washout are independent of task specifications

We tested for retention of the newly learned motor control policy by comparing the last adaptation block of the *Learning* period with the first adaptation block of the *Retention* period after a 30-minute break. Subjects successfully retained or even showed additional gains in motor performance after the break with significantly lower angular errors in the first retention block for both *Reaching* (t_(65)_ = 3.35, *p* = 0.001; Fig. 2e) and *Curling* (t_(65)_ = 2.3, *p* = 0.023; Fig. 2f). Of note, subjects did not receive performance-dependent feedback in the *Retention* period, regardless of reinforcement condition.

In the washout block (Fig. 1), mean angular errors were lower in *Reaching* than in *Curling* (washout *Reaching*: 2.70°, *Curling*: 7.55°; t_(65)_ = 18.30, *p* < 0.001). On the other hand, the magnitude of baseline-to-washout changes in angular errors was comparable across tasks (*Reaching*: 0.84° ± 1.16°, *Curling*: 1.26° ± 2.07°; t_(65)_ = 1.49, *p* = 0.14). Hence, these findings further support the notion that the temporal profiles of adaptation were similar for both tasks, with the availability of *within-trial* learning resulting in superior performance in the *Reaching* task.

### Reinforcement selectively informs adaptation in *within-trial* learning

**Fig. 3:**
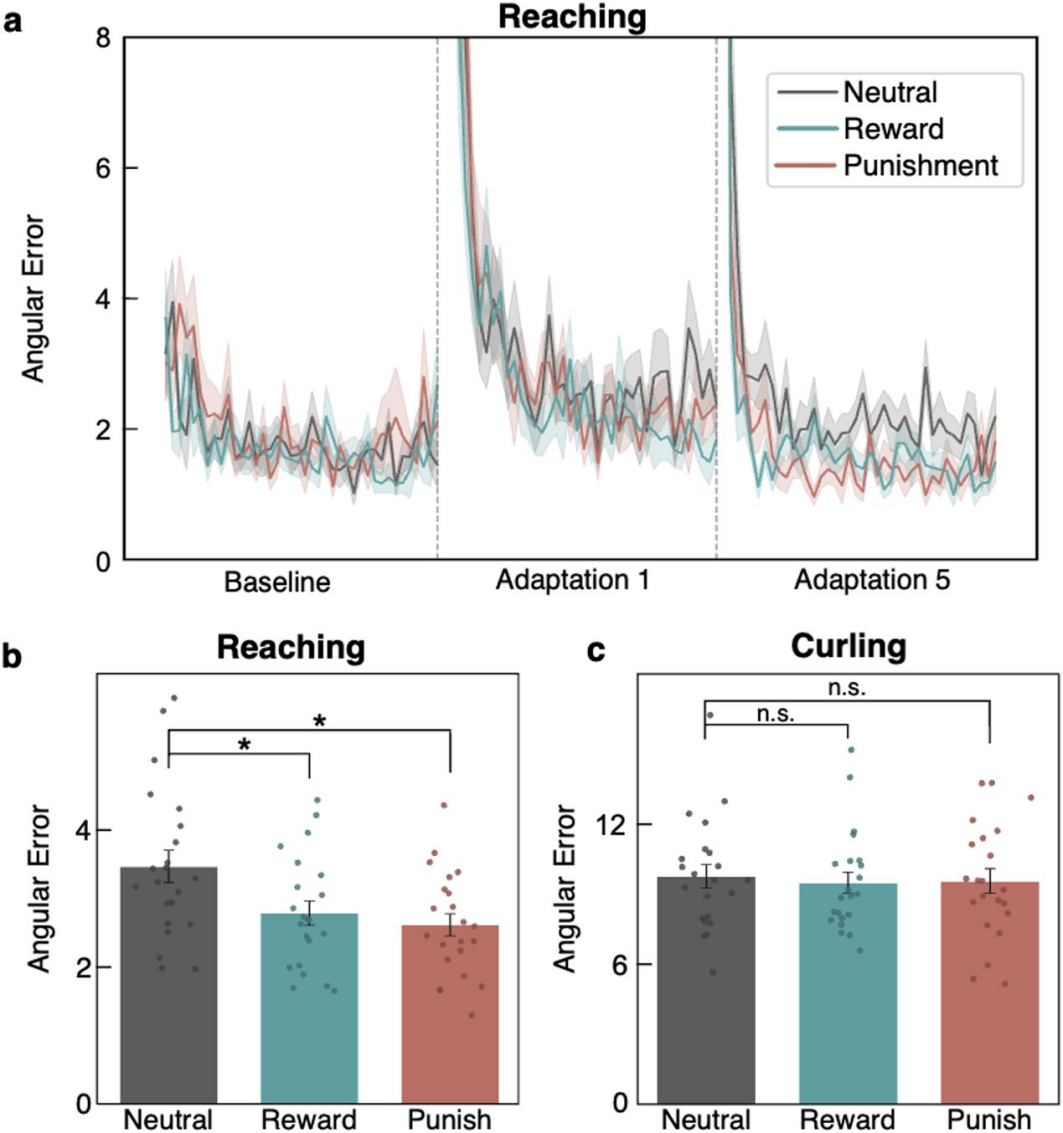
Task‑dependent role of reinforcement for adaptation. **(a)** Mean angular errors (± SEM) across reinforcement conditions are depicted for baseline trials, the first adaptation (Adaptation 1) and the last adaptation (Adaptation 5) block for the *Reaching* task. While angular errors were highly similar across reinforcement conditions early on (Adaptation 1), reinforcement-dependent differences in angular errors emerged over time (Adaptation 5). **(b)** During *Reaching*, performance-dependent reward (*green*) and punishment (*red*) significantly reduced angular errors compared to neutral feedback (*grey*). **(c)** Conversely, no significant reinforcement effects were detected during *Curling*. Hence, performance-dependent reinforcement feedback via both monetary reward and punishment enhanced motor adaptation only when *within-trial* learning was possible. (n.s. “not significant”, * *p* < 0.05, ** *p* < 0.01, and *** *p* < 0.001).

To investigate whether reinforcement differentially modulated *within-trial* and *between-trial* learning, we first examined the task x condition interaction in the mixed-effects model, which indicated that reinforcement effects depended on the task (task x condition interaction: χ^2^_(2)_ = 9.60, *p* = 0.008). Subsequent task-specific ANOVAs showed a significant main effect of reinforcement for the *Reaching* (F_(2,63)_ = 5.45, *p* = 0.007, generalised η^2^ = 0.15; Fig. 3b) but not for the *Curling* task (F_(2,63)_ = 0.079, *p* = 0.92; Fig. 3c). Thus, reinforcement led to better performance in the *Reaching* task only. Post-hoc tests revealed that subjects performed significantly better in the reward (t_(42)_ = 2.49, *p* = 0.023, FDR-corrected) and punishment (t_(42)_ = 3.12, *p* = 0.008, FDR-corrected) conditions compared to the neutral condition. ANOVAs, including the mean angular error of each adaptation block separately (Adaptation 1–5), revealed time-dependent effects of reinforcement on motor adaptation: For the first adaptation block (Adaptation 1), no significant reinforcement effect was detected (F_(2,63)_ = 20.24, *p* = 0.78). In the following adaptation blocks, subjects performed significantly better with punishment (*p* < 0.05 in every adaptation block, FDR-corrected) or reward (*p* < 0.05 in every adaptation block, FDR-corrected) compared to the neutral condition (Supplementary Fig. S2). No significant differences between reward and punishment were detected for any adaptation block (*p* > 0.05 in every adaptation block).

### Early variability primarily informs between-trial learning

To assess whether motor variability upon introduction of the visuomotor rotation was associated with subsequent motor adaptation, we quantified early motor variability via the standard deviation of the first 5 trials within each adaptation block.

**Fig. 4:**
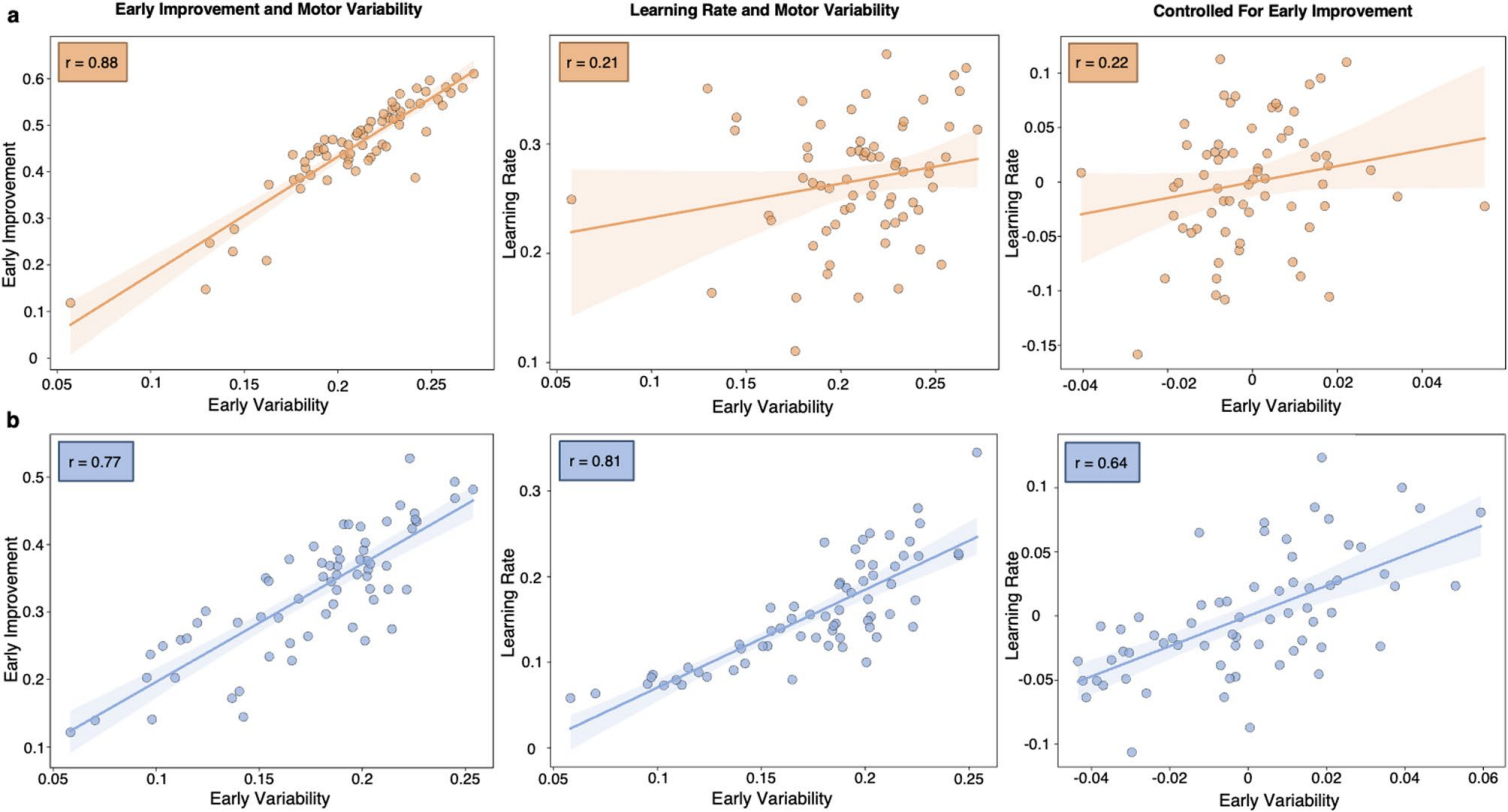
Task‑dependent role of early motor variability for adaptation. The relationships between early motor variability and (i) early improvement in performance (angular error between trial 1 – trial 5), (ii) model‑based learning rates, and (iii) learning rates after statistically controlling for early improvement (via partial correlations) are depicted for (**a**) the *Reaching* task and (**b**) the *Curling* task. **(a)** In *Reaching*, early variability was associated with early improvement in performance but not learning rates, indicating that it largely reflected initial error reduction. In line with this notion, no association between early variability and learning rate was observed when controlling for early improvement. **(b)** In *Curling*, early variability also correlated with early improvement. However, early variability was also positively and significantly related to the model‑based learning rate, even when controlling for early improvement. Hence, higher degrees of early variability were associated with better subsequent learning. Conceptually, early variability in *Curling* may reflect planning noise, potentially representing exploration of the motor output space benefiting subsequent between-trial learning^23^.

For both tasks, early motor variability was significantly correlated with early improvements in performance (angular error trial 1 – trial 5), demonstrating that early error reduction substantially shaped early variability in both tasks (*Curling*: *r* = 0.77, *p* < 0.001; *Reaching*: *r* = 0.88, *p* < 0.001; Fig. 4).

To test whether early motor variability influenced subsequent adaptation, we examined its influence on the model-based learning rates, representing learning across the whole block. Here, no significant association between early variability and learning rates was observed for the *Reaching* task (*r* = 0.21, *p* = 0.098; Fig. 4a). Conversely, a significant positive correlation was found for the *Curling* task (*r* = 0.81, *p* < 0.001; Fig. 4b), with subjects featuring pronounced early variability also showing higher learning rates. To assess whether this association was largely driven by the degree of early improvements we computed partial correlation analyses controlling for early improvements in performance. The association between early variability and learning rates remained significant for *Curling* (*r* = 0.64, *p* < 0001; Fig. 4b) and non-significant for *Reaching* (*r* = 0.22, *p* = 0.07; Fig. 4a) even when controlling for early improvement. Thus, for *Curling*, early motor variability captured variance positively associated with subsequent adaptation independent of initial learning. This finding is nicely in line with the notion that early variability may reflect planning noise, potentially representing exploration of the motor output space benefiting between-trial learning^23,25^.

Moreover, early variability was significantly higher in the *Curling* task compared to the *Reaching* task. This difference was present both within each adaptation block (*p* < 0.05, Supplementary Fig. S3) and across all adaptation blocks (V = 441, *p* < 0.001; Supplementary Fig. S3). While we found a significant main effect of reinforcement on early motor variability in the *Curling* task (F_(2,63)_ = 3.20, *p* = 0.047, generalised η^2^ = 0.092), none of the post-hoc tests between conditions were significant after FDR-correction (punishment vs. reward t_(42)_ = 2.10, *p* = 0.059, FDR-corrected; punishment vs. neutral: t_(42)_ = 2.27, *p* = 0.059, FDR-corrected; reward vs. neutral: t_(42)_ = 0.19, *p* = 0.87). No reinforcement main effects on early variability were detected for the *Reaching* task (F_(2,63)_ = 0.38, *p* = 0.69).

## Discussion

Motor adaptation is thought to involve at least two distinct learning processes: *within-* and *between-trial* adaptation. *Between-trial* adaptation arises from updates to the motor plan based on previous errors^9^. This notion is supported by evidence of adaptation occurring in the absence of continuous visual feedback^8^. The importance of motor planning for adaptation is further highlighted by its modulation through factors like perceptual recalibration or disruption of preparatory neural activity in the motor cortex^28,29^. In parallel, *within-trial* adaptation arises from the online correction of ongoing movements driven by continuously available SPEs^6,7^.

Notably, the precise contribution of online correction to motor adaptation remains an ongoing scientific debate. While seminal work by Tseng and colleagues suggested that the additional role of *within-trial* learning may be limited compared to *between-trial* learning alone^6^, other data showed that *within-trial* control mechanisms are fundamentally adaptive and mechanistically differ from *between-trial* planning^7,24,30,31^. This raises the question of how *within*- and *between-trial* learning interact to inform visuomotor adaptation.

**Fig. 5:**
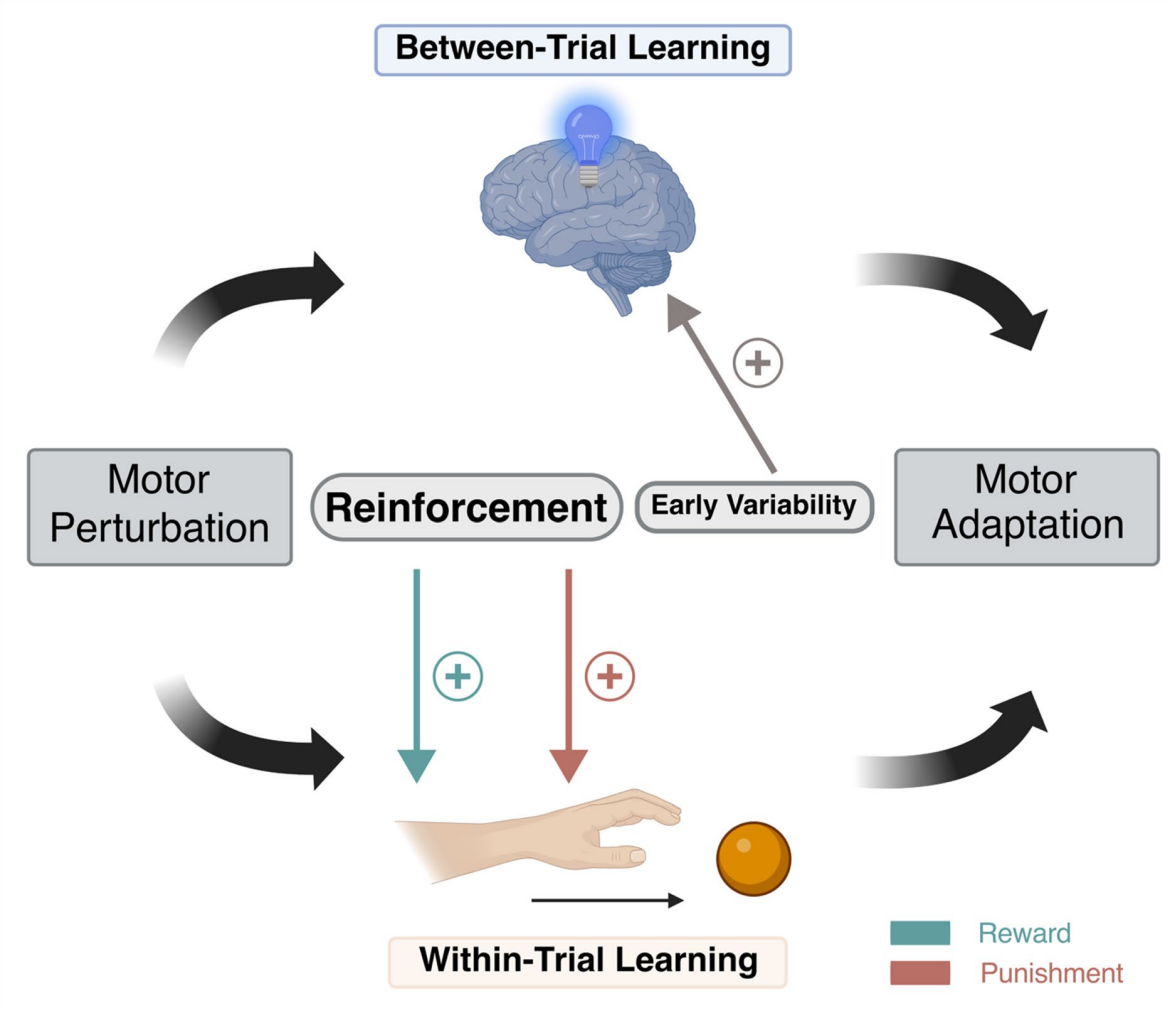
Conceptual framework summarising the role of *within*- and *between*-*trial* learning on motor adaptation. Motor adaptation in response to a perturbation arises from at least two distinct processes: *between-trial* learning driven by motor planning and *within-trial* learning driven by online correction of ongoing movements. Our current findings suggest a functional dissociation: reinforcement (reward depicted in *green* and punishment depicted in *red*) enhanced *within-trial* but not *between-trial* learning. Conversely, early motor variability primarily benefited *between-trial* learning in the *Curling* task. Such beneficial “planning noise” may reflect the exploration of the motor output space potentially informing the optimisation of motor control policies between trials.

Our findings demonstrate that substantial motor adaptation can be driven by both *between-trial* updates to motor planning (*Curling*) and a combination of *between-* and *within-trial* adaptation (*Reaching*) (see Figs. 2 and 5). Of note, both tasks elicited equivalent hallmarks of adaptation: error reduction over time (Fig. 2a), savings reflected by faster re-adaptation^32,33^ and retention^34–37^ (Fig. 2e and f). The presence of these features in the *Curling* task underscores the capacity of *between-trial* learning arising from motor plan adjustments to guide motor adaptation. In direct comparison, the additional availability of online correction via *within-trial* information resulted in higher learning rates and lower angular errors during *Reaching* (Fig. 2c and d), aligning nicely with previous work demonstrating that online correction processes may maximise adaptation efficiency^7,24^. Consistent with this interpretation, *Reaching* showed pronounced improvements in performance when comparing angular errors quantified at an early reference point (“release point” of *Curling*) to the angular errors at peak velocity, supporting the notion that *within-trial* performance gains in *Reaching* were driven by online corrections of ongoing movements (see Supplementary Fig. S5).

Interestingly, despite this clear performance gap, the temporal profiles of adaptation were highly similar across both tasks, with performance plateauing after a similar number of blocks. This finding suggests that while online correction provides a powerful additive benefit, it may not fundamentally alter the time course of *between-trial* adaptation. This observation is consistent with Tseng et al. (2007) who argued that trial-to-trial processes are primarily driven by initial SPEs, available in both of our tasks^6^. Given that both *within-* and *between-trial* learning led to successful visuomotor adaptation, the question arises whether each of these processes may be distinctly susceptible to factors modulating adaptation, such as reinforcement.

Reinforcement is known to readily modulate motor adaptation^10,12–15^. Recent evidence links reinforcement signals affecting motor control to cerebellar-basal ganglia circuits^18^. Notably, these circuits are known to be crucial for *within-trial* online correction^6,38,39^, leading to the hypothesis that reinforcement primarily modulates adaptation through *within-trial* learning. In line with this notion, we found that reinforcement significantly improved performance during *Reachin*g (Fig. 3) but not *Curling*. Thus, cerebellar-basal-ganglia-loops (sensorimotor feedback loops) might fundamentally shape motor adaptation in the presence of reinforcement^18,19^. Further support for this hypothesis derives from recent findings outlining that reward may increase feedback control to enhance the online correction of movement errors^40,41^. Conversely, reinforcement did not modulate performance in the *Curling* task. At first glance, this appears to be at odds with seminal findings by Codol et al. (2020), who reported that reinforcement modulated action selection. Then again, *between-trial* adaptation in our *Curling* task was markedly distinct from the concept of action selection, which can precede and co-occur with movement execution^17^. By decoupling these processes, our findings suggest that the planning of a movement trajectory without online correction cannot be easily modulated by reinforcement. This dissociation helps explain previous contradictory findings regarding the role of reinforcement in motor adaptation. In particular, punishment has been shown to enhance the *initial learning*^14^, to only affect the *final extent* of learning^15^, or to have *no effect at all*^10^. Conversely, reward has been reported to improve *both learning rate and extent*^12^ or to have *no immediate* effect^10^. Furthermore, the well-documented benefit of reward on *retention*^10,14^ is not always replicated^15^. Our current findings suggest a potential explanation for these inconsistent results: reinforcement might differentially impact *within-trial* and *between-trial* learning, which are distinctly represented in the mentioned studies, thus leading to different results depending on the given task. For example, given our current findings, it may seem far less surprising that Roth and colleagues did not replicate the beneficial effect of reward on retention given that their task more heavily relied on *between-trial* adaptation^15^ which was not modulated by reward here (Fig. 3).

Besides reinforcement, other task-dependent factors, such as distinct roles of early motor variability, may additionally shape motor adaptation. The theoretical framework by Dhawale and colleagues (2017) dichotomises motor variability to reflect either central “planning noise” relevant for motor adaptation or peripheral “execution noise” holding no meaningful implications^23^. However, whether motor variability constitutes detrimental noise or a valuable teaching signal remains a topic of ongoing debate^20,22,42^. Our current results provide support for the notion that the availability of *within-trial* learning critically influences the functional role of motor variability.

Specifically, we found that early motor variability correlated with learning rates when adaptation solely relied on *between-trial* updates to motor planning (*Curling*, Fig. 4b). Of note, this relationship was preserved when controlling for early performance improvement in *Curling* (Fig. 4b). Hence, in *Curling*, early variability not only reflected initial improvement in behaviour but was also indicative of subsequent adaptation. From a mechanistic perspective, higher early variability may partly reflect beneficial planning noise or motor exploration^23^ informed by target errors^8^. Support for this notion derives from recent work reporting that “planned movement variability” meaningfully informed exploratory behaviour rather than reflecting undifferentiated noise^25^ and was positively associated with learning rates^43^. Notably, in the *Reaching* task, early variability was not associated with learning rates. Here, the availability of online correction may “dampen” the informational value of early variability, as the dense stream of *within-trial* error information may allow for errors to be corrected on-the-fly and hence ameliorate instructive target errors.

Taken together, our findings suggest that the modulatory influences of reinforcement and motor variability may depend on the degree to which *within-* and *between-trial* learning contribute to adaptation in a given task (Fig. 5).

A probable limitation of our current findings may arise from a conceivable difference in difficulty between tasks, reflected by higher learning rates and lower angular errors during *Reaching*. Hence, one might argue that the observed differences in adaptation and susceptibility to reinforcement between tasks are partly attributable to a disparity in difficulty. Then again, the similarity of the temporal trajectories and presence of adaptation hallmarks for both *Reaching* and *Curling* render a substantial bias induced by potential differences in task difficulty unlikely. Moreover, while the *Curling* task was designed to prevent online corrections of ongoing movements, we cannot fully exclude that *within-trial* learning may have occurred, albeit to a far lesser degree. Finally, while our single-rate model successfully captured adaptation and enabled comparisons across tasks, it did not allow for a model-based distinction of *within-* and *between-trial* processes. Future work combining suitable experimental designs with more sophisticated models may help to further delineate whether reinforcement selectively targets distinct processes contributing to motor adaptation.

In this study, we sought to disentangle the relative contributions of *between-trial* and *within-trial* learning to motor adaptation by contrasting a task that primarily relied on trial-to-trial updating of a ballistic motor plan with a task that, in addition, permitted continuous online corrections of ongoing movements. Our data demonstrated that successful adaptation and retention can arise from both *within*- and *between-trial* learning. Importantly, reinforcement-dependent facilitation of adaptation via reward and punishment critically relied on the availability of online correction, highlighting the seminal role of *within-trial* SPE-processing in mediating reinforcement effects. In contrast, motor variability exclusively benefited adaptation driven by *between-trial* learning. Here, early variability may represent beneficial “planning noise” potentially reflecting the exploration of the motor output space, which may inform the subsequent optimisation of motor plans. In summary, our current findings expand our mechanistic understanding of motor adaptation by emphasizing that the modulatory influence of reinforcement and motor variability may depend on the distinct contribution of *within-* and *between-trial* learning to adaptation in the tasks at hand (Fig. 5).

## Materials and methods

### Participants

Sixty-six right-handed subjects (*n* = 66, 39 female, age = 25.4 +- 1.97 years) performed two distinct visuomotor adaptation joystick tasks on two different days (> 1 day apart). Reinforcement differed between participants who either received performance-dependent reward or punishment, or neutral feedback (*n* = 22 each) in line with previous work^14^. All participants provided written informed consent before participation. The study was approved by the Human Research Ethics committee at the University Hospital Cologne and was carried out under the Declaration of Helsinki.

## Experimental setup

Participants were seated in a chair with their right arm resting on a cushioned armrest and their right hand engaging with a 2-axis analogue custom joystick, which could be deflected in any planar direction while providing continuous X and Y position signals to control cursor movements. Joystick movements were sampled at a rate of 1000 Hz via a Biopac^®^ MP36-Interface (Biopac Systems Inc., 42 Aero Camino, Goleta, CA 93117, USA). The seating position was individually adjusted to align arm and shoulder positions for all subjects. The hand holding the joystick was covered to prevent visual perception of the hand or joystick position (Fig. 1c).

### Task design

Subjects performed two visuomotor adaptation tasks which are referred to as the *Reaching* and *Curling* tasks. The order of task performance was counterbalanced across sessions.

In the *Reaching* task, trials terminated when the cursor reached the target, requiring centre-out movements that offered continuous control and allowed for online corrections of cursor movements throughout the whole trial (Fig. 1a). Subjects were instructed to perform a single, smooth movement towards the leftward target as quickly as possible, to avoid overshooting, and to end up as closely to the target as possible. The full range of motion from the starting point to the target (in the *Reaching* task) mapped to 12 cm of forearm and hand movements. Given that the *Reaching* task permitted online corrections of the ongoing movement, it was assumed to enable *within-trial* as well as *between-trial* adaptation.

In the *Curling* task, each trial was split into an initial, joystick-controlled “launch phase” and a subsequent “autonomous phase”. Participants were instructed to initiate each trial from the starting position and execute a single, quick shooting movement towards the target. During the initial launch phase, the cursor was controlled by the joystick position. At a radial distance of 8 mm from the starting position, the “release point” was reached. From the release point, the cursor autonomously moved in a straight line at a constant speed of approximately 21 cm/s along the initiated movement direction. During this “autonomous phase”, participants could no longer influence the cursor which continued to move until it reached the horizontal coordinate of the target. In the absence of online correction, *Curling* performance was therefore determined by the initial movement direction, akin to the homonymous winter sport. Improvements in *Curling* hence relied on *between-trial* rather than *within-trial* adaptation. Exemplary single-subject trajectories for both tasks are shown in Supplementary Fig. S4 to further illustrate differences between both tasks.

The experimental design featured alternating 40-trial blocks. During adaptation blocks, a systematic 40° counterclockwise visuomotor rotation was applied, while during de-adaptation blocks, this rotation was removed (Fig. 1d).

Each session included a brief initial familiarisation period (25 trials) for participants to acquaint themselves with the task setup before the experiment. The experiment comprised a *Learning* period (400 trials) and a *Retention* period (160 trials). The *Learning* period began with a baseline block of 40 unrotated trials, followed by alternating blocks of rotated (adaptation) and unrotated (de-adaptation) trials, each comprising 40 trials. After a 30-minute break, subjects completed the *Retention* period. The *Retention* period comprised two blocks of 40 rotated trials (adaptation) and 40 unrotated trials (de-adaptation, washout) performed in alternating order. Notably, the *Retention* period began with rotated (adaptation) trials to assess the impact of the 30-minute break on adaptation.

Task performance was quantified via the angular error, defined as the angle (°) between the optimal (start to target) and actual (start to cursor at a reference point) movement vectors. For the *Reaching* task, the reference point was defined as the point of peak cursor velocity in line with previous work^44–46^. Thus, in the *Reaching* task, angular errors captured a combination of *within-* and *between‑trial* adaptation, as subjects could continuously correct their movements online (see Supplementary Fig. S4). For the *Curling* task, the reference point was defined as the “release point”, after which subjects could no longer control the cursor via joystick movements. Hence, in the *Curling* task, trial‑wise angular errors primarily reflected *between‑trial* adaptation arising from updates to the planned initial movement direction.

During the *Learning* period, group-specific reinforcement was applied. Participants in the reward group started with zero points and received 0–10 points per trial depending on their performance. Participants in the punishment group began with an endowment of 4000 points and lost 0–10 points per trial based on their performance. The gained or lost points of each trial were displayed after the trial ended. Participants in the reward and punishment groups were paid based on the total number of points accumulated throughout the experiment (10 points = 0.062€). Neutral group participants received no trial feedback and were paid a fixed amount of 13€ per task. Importantly, participants never received feedback during the *Retention* period, regardless of the reinforcement condition (Fig. 1d).

### Power analysis

An a priori power analysis was conducted using G*Power for a repeated-measures ANOVA with a within–between interaction (two within subject tasks, three between-subject reinforcement conditions and repeated measures across tasks and blocks). Assuming a medium effect size of $\:f=0.25$, an alpha level of 0.05, and a desired power of 0.95, the analysis indicated a required total sample size of 66 participants. Our sample of subjects was recruited to meet this requirement, ensuring that the study was adequately powered to detect interaction effects of the desired magnitude.

### Statistical analyses

We employed both model-free and model-based analyses of trial-wise angular errors to probe for motor adaptation.

Initially, extreme outliers (larger than 60°) were removed. A sliding-window Hampel filter (window size = 6 trials) was applied to identify and replace additional outliers with the median of the window. Of note, the first five trials of each block were excluded from this sliding-window approach. Across all subjects and conditions, 7.59% of trials were identified as outliers (M = 42.50 trials per subject per task, SD = 10.14, range 23–67 trials). Outlier rates were slightly higher in *Reaching* (8.56%, M = 47.91 ± 7.51 trials per subject) than *Curling* (6.62%, M = 37.09 ± 7.29 trials per subject). Of note, outlier rates were comparable across reinforcement conditions (neutral: 7.53%, reward: 7.35%, punishment: 7.90%), rendering a bias introduced by outlier trials highly unlikely. Assumptions for parametric testing were assessed prior to analyses. Normality of data distribution was confirmed by visual inspection of Q-Q plots, and homogeneity of variances was checked using Levene’s tests. In case of a violation of these assumptions, appropriate non-parametric tests were utilised.

For model-based analyses, we employed a single-rate state-space model to compute learning rates for each block^14^. We employed a single-rate state-space model to estimate block-wise learning rates, as it (i) converged reliably for both tasks (see goodness-of-fit diagnostics below), (ii) provided interpretable estimates directly comparable between *Reaching* and *Curling*, and (iii) aligned with prior work on reinforcement-based motor adaptation^14^. Before fitting the model, the decimal logarithm of trial-wise angular errors of rotated trials in adaptation blocks was standardised to the median baseline performance within each subject (median of the baseline block) to remove biases in the fitting process due to interindividual differences in the range of angular errors. We then fitted the *single-rate state-space model*,

$$\:{\mathrm{y}}_{\mathrm{n}}={-\mathrm{z}}_{\mathrm{n}}^{\mathrm{t}}$$

$$\:{\mathrm{z}}_{\mathrm{n}+1}^{\mathrm{t}}=\mathrm{A}{\mathrm{z}}_{\mathrm{n}}^{\mathrm{t}}+\mathrm{B}({\mathrm{r}}_{\mathrm{n}}-{\mathrm{z}}_{\mathrm{n}}^{\mathrm{t}})$$

in which $\:{\mathrm{y}}_{\mathrm{n}}$ represents the angular hand direction relative to the target on trial $\:\mathrm{n}$. $\:\mathrm{z}$ is the state of the learner on trial $\:\mathrm{n}$, signifying the current estimated visuomotor rotation linked to the target $\:\mathrm{t}$. $\:{\mathrm{r}}_{\mathrm{n}}$ represents the visuomotor rotation applied during trial $\:\mathrm{n}$. $\:{\mathrm{r}}_{\mathrm{n}}-{\mathrm{z}}_{\mathrm{n}}^{\mathrm{t}}$ denotes the angular error relative to the target. Within this model, the learning rate $\:\mathrm{B}$ governs how much of the cursor error $\:({\mathrm{r}}_{\mathrm{n}}-{\mathrm{z}}_{\mathrm{n}}^{\mathrm{t}})$ is subject to adaptation, while the decay parameter $\:\mathrm{A}$ describes the rate at which the state’s estimated visuomotor mapping $\:{(\mathrm{z}}_{\mathrm{n}}^{\mathrm{t}})$ is forgotten^14^. Successful model fit was achieved for all subjects using the minimise-function of the Scipy library^47^ with the SLSQP-method based on least-squares errors.

Single-rate state-space models converged for all blocks in both tasks (*Reaching*: 924/924 successful optimisations; *Curling*: 924/924 successful optimisations). For the *Reaching* task, overall fit quality was R² = 0.6788 ± 0.2098 (mean ± SD; median = 0.6788), RMSE = 0.0506 ± 0.0159 (median = 0.0506), observed and predicted angular errors were significantly correlated (*r* = 0.87 ± 0.08, *p* < 0.001). For the *Curling* task, overall fit quality was R² = 0.3448 ± 0.1595 (median = 0.3448), RMSE = 0.0922 ± 0.0182, and observed and predicted angular errors were correlated significantly (*r* = 0.60 ± 0.13, *p* = 0.003).

### Linear mixed-effects model

To analyse adaptation across tasks and test for main effects and interactions between experimental factors, a linear mixed-effects model was fitted using the angular error data including all adaptation trials from the *Learning* period. Fixed effects comprised task (*Reaching* vs. *Curling*), reinforcement condition (neutral, reward, punishment), block (adaptation blocks 1–5), and trial number within block (trial), which was entered as a mean‑centred continuous predictor to capture within‑block adaptation. Interaction terms between task, reinforcement condition, block, and trial were included to assess task‑ and reinforcement‑dependent differences in adaptation across blocks and tasks. Random intercepts for subjects and random slopes for trial per subject were included to account for individual differences in overall performance levels, while retaining a maximal random-effects structure. Model assumptions (normality and homoscedasticity of residuals) were checked by visual inspection of Q-Q plots and by computing Levene’s tests. Fixed effects were evaluated using Type III F‑tests from the mixed-model ANOVA. In case of violations of model assumptions, corresponding effects were followed up with the appropriate non‑parametric tests reported in the Results section.

### Effects of motor adaptation within and across blocks in both tasks

To test whether subjects exhibited successful adaptation within and across blocks, we first examined the effect of block (adaptation blocks 1–5) in the mixed-effects model, which captured across-block changes in averaged trial-wise angular errors while accounting for task, reinforcement condition and subject-level random effects. A significant main effect of block was followed up by post-hoc tests. Within each task, we compared consecutive adaptation blocks (1–5) using FDR-corrected paired t-tests for the model-free measure (mean angular error per block) and Wilcoxon signed-rank tests for the model-based measure (block-wise learning rate). To assess successful within-block adaptation, we assessed the main effect of trial in the mixed-effects model and additionally compared early versus late trials within each adaptation block (mean angular error of the first five vs. last five trials in line with previous work^12,48,49^) using Wilcoxon signed-rank tests for both tasks.

### Task comparison

To compare performance between the *Reaching* and *Curling* tasks, we checked for a main effect of task in the mixed-effects model. We then performed post-hoc paired-samples t-tests in the model-free and model-based data on subject-level summary measures. For the model-free analysis, the mean angular error of the adaptation blocks of the *Learning* period was compared between the two tasks. Similarly, for the model-based analysis we compared the mean learning rate of the adaptation blocks of the *Learning* period between tasks.

To empirically verify that the *Reaching* task permitted *within-trial* online corrections and the *Curling* task did not, we analysed cursor trajectories and movement times for both tasks. For each trial, movement directions were computed from the time series of sampled cursor positions. A substantial change in movement direction was assumed when the angular error calculated for two successive samples exceeded 5°. In addition, we quantified the duration of active motor control in each task separately (control time). Both measures were compared between tasks via paired-samples t-tests (see Supplementary Fig. S4).

### Comparison of angular errors at matched reference points

To enable a direct comparison of initial movement directions between *Reaching* and *Curling*, we computed angular errors in the *Reaching* task at the same “release point” (8 mm distance from the starting position) used to determine angular errors in *Curling* (see Fig. 1). We compared mean angular errors of initial movement directions using a paired samples t-test between *Reaching* and *Curling* (see Supplementary Fig. S5). The original angular error for *Reaching* (quantified at peak velocity) was retained for all primary analyses, as it allowed to capture *within-trial* online corrections of the movement trajectory^45,46,50^.

### Retention effects and washout

To test for retention effects, we used paired *t*-tests to compare the mean angular errors of the last adaptation block in the *Learning* period with the first retention block (Retention 1; Fig. 2e&f). Moreover, we compared the mean angular errors in the final washout block across tasks using paired *t*-tests. Finally, task specific changes in performance from the baseline to the washout period were computed (washout − baseline) and compared across tasks using paired *t*-tests.

### Reinforcement effects

Reinforcement effects were assessed via the main effect of condition (neutral, reward, punishment) and the task x condition interaction. Given a significant task x condition interaction, we followed-up with separate one-way ANOVAs to test for reinforcement effects on the performance measure in each task with the between-subject factor condition on the mean angular error of each subject across all adaptation blocks of the *Learning* period. Furthermore, we performed ANOVAs for each adaptation block of the *Learning* period separately with the between-subjects factor condition to assess reinforcement effects within each block. Post-hoc independent *t*-tests between conditions were conducted and FDR-corrected to account for multiple comparisons.

### Motor variability and learning rate analysis

To assess the influence of early motor variability on subsequent adaptation we computed correlations between the early motor variability (standard deviation of the angular error of the first five trials of each adaptation block) with (i) early improvement (angular error trial 1 – trial 5) and (ii) the model-based learning rates during the *Learning* period. To quantify the contribution of early variability to subsequent adaptation beyond initial error reduction, we additionally computed partial correlations between early variability and learning rates while statistically controlling for the degree of early improvement (see Fig. 4).

To compare motor variability between tasks, we computed Wilcoxon signed-rank tests using the mean standard deviation of the first five trials across all five adaptation blocks of the *Learning* period for each subject (Fig. 4c). Additionally, we compared the variance of the first five trials in each adaptation block of the *Learning* period separately between *Reaching* and *Curling* (Supplementary Fig. S3). Furthermore, we performed ANOVAs with the between-subject factor condition to investigate reinforcement effects on mean early motor variability per subject. Post-hoc independent *t*-tests were performed, and *p*-values were FDR-corrected for multiple comparisons.

## Supplementary Information

Below is the link to the electronic supplementary material.

Supplementary Material 1

## Data Availability

Code is available in a public repository at the following link: bit.ly/4pFMU9U. Data are available from the corresponding author upon reasonable request.
