# Supplementary material for: Reinforcement drives within- not between-trial motor adaptation
Source: Sci Rep. 2026 Apr 4;16:11605. doi: 10.1038/s41598-026-45293-8 (PMC13056898; doi:10.1038/s41598-026-45293-8)
Supplement: Supplementary file 1 — Supplementary Material 1 [file 41598_2026_45293_MOESM1_ESM.docx]

**Supplementary Material**

*
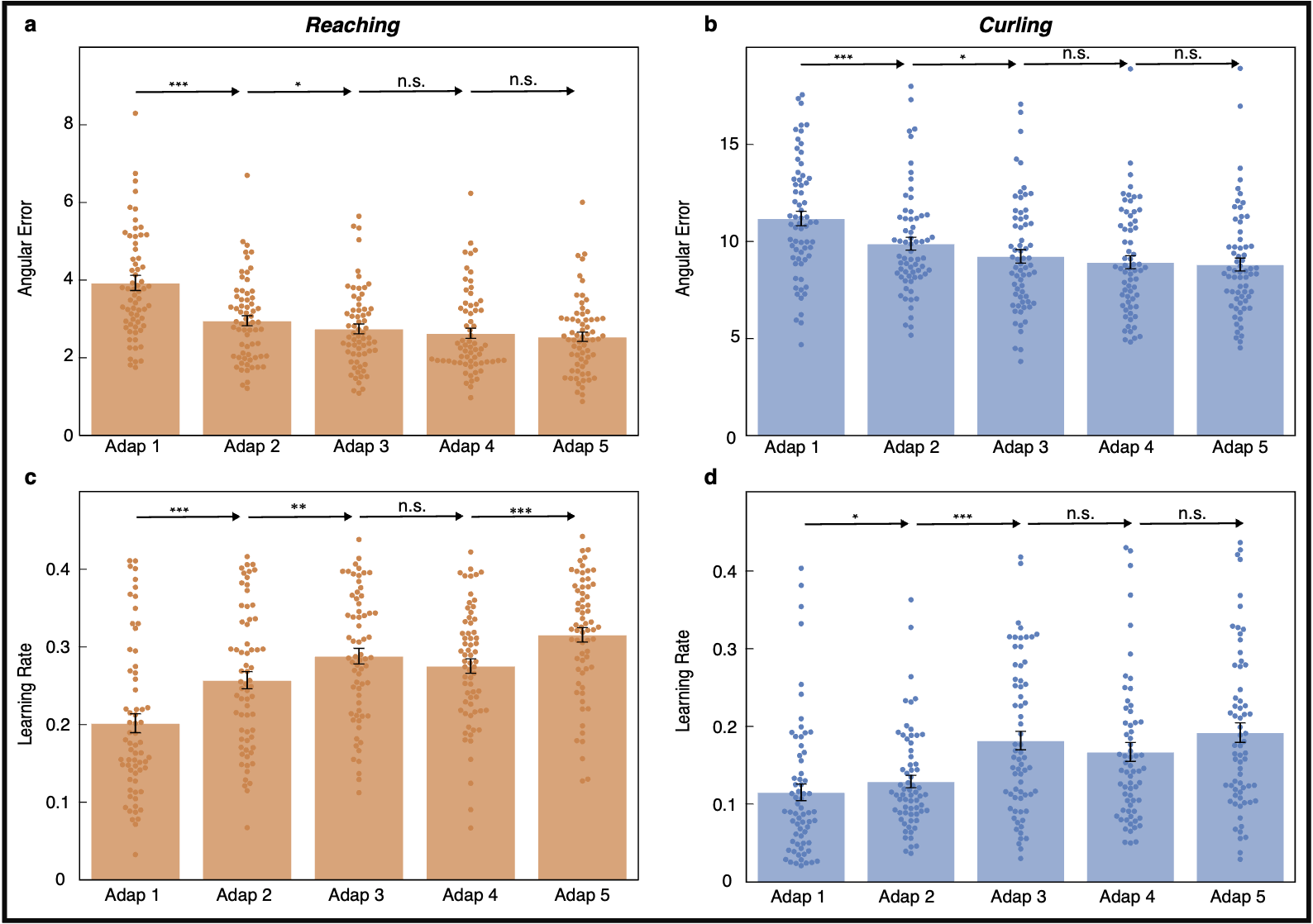
*

**S1:** **Adaptation performance and learning rates during the Learning period.**

**a** Mean angular errors in the *Reaching* task progressively decreased across blocks, demonstrating successful adaptation. **b** The *Curling* task showed a similar progressive decrease in mean angular errors over time. **c** Correspondingly, model-based learning rates for the *Reaching* task increased across the initial blocks, indicating accelerated adaptation with repeated exposure to the visuomotor rotation. **d** Learning rates in the *Curling* task also increased across blocks before reaching a plateau. Overall, the *Reaching* task showed consistently lower angular errors and higher learning rates, highlighting the contribution of online correction to faster and more complete adaptation. (n.s.: not significant, * *p* < 0.05, ** *p* < 0.01, and *** *p* < 0.001)

*
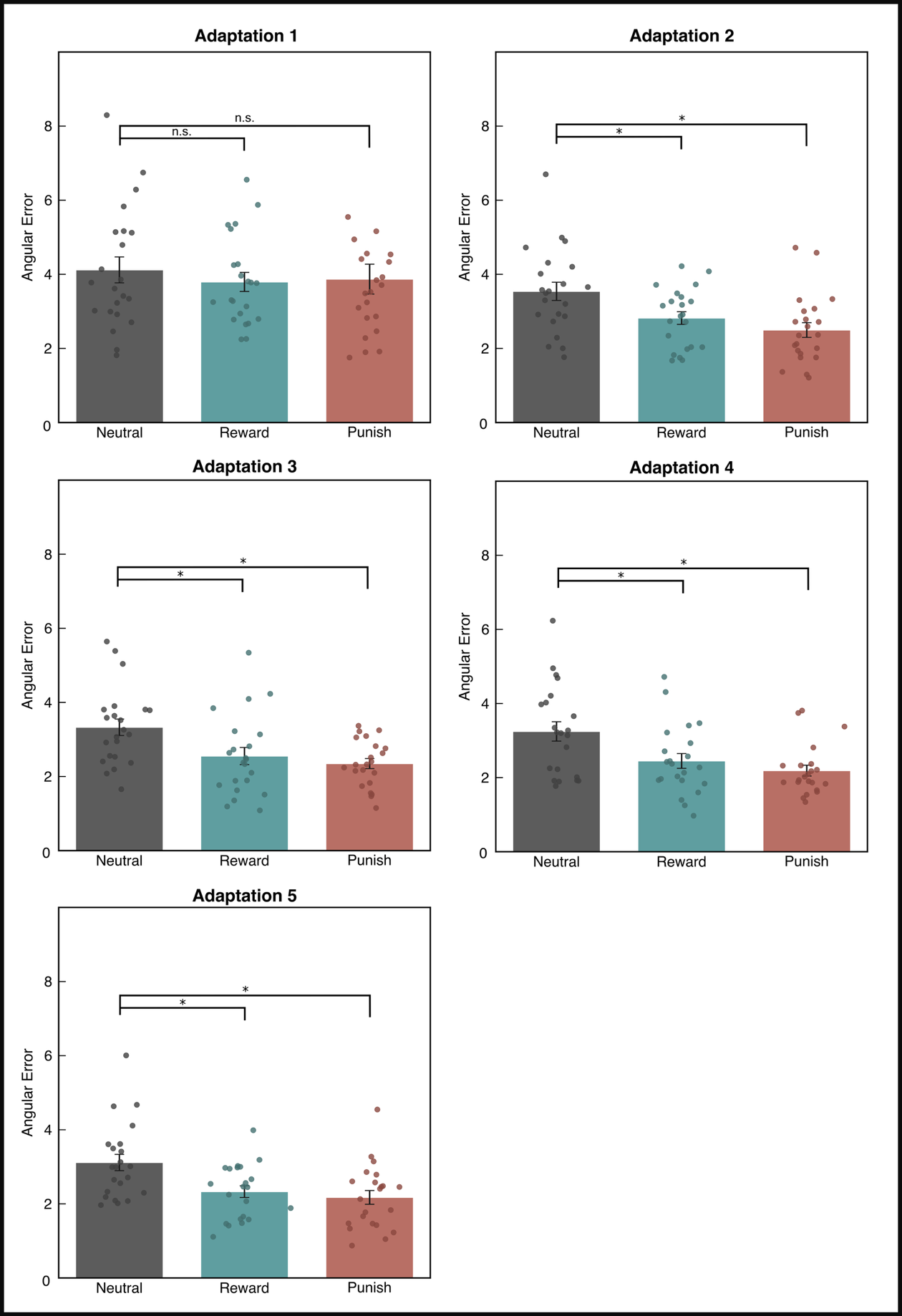
*

**S2:** **Reinforcement effects in the *Reaching* task.**

The impact of reinforcement feedback on motor adaptation in the *Reaching* task unfolded over time. In the first adaptation block, performance was comparable across all conditions. However, from the second block onwards, both reward and punishment significantly improved performance relative to the neutral condition (* *p* < 0.05).


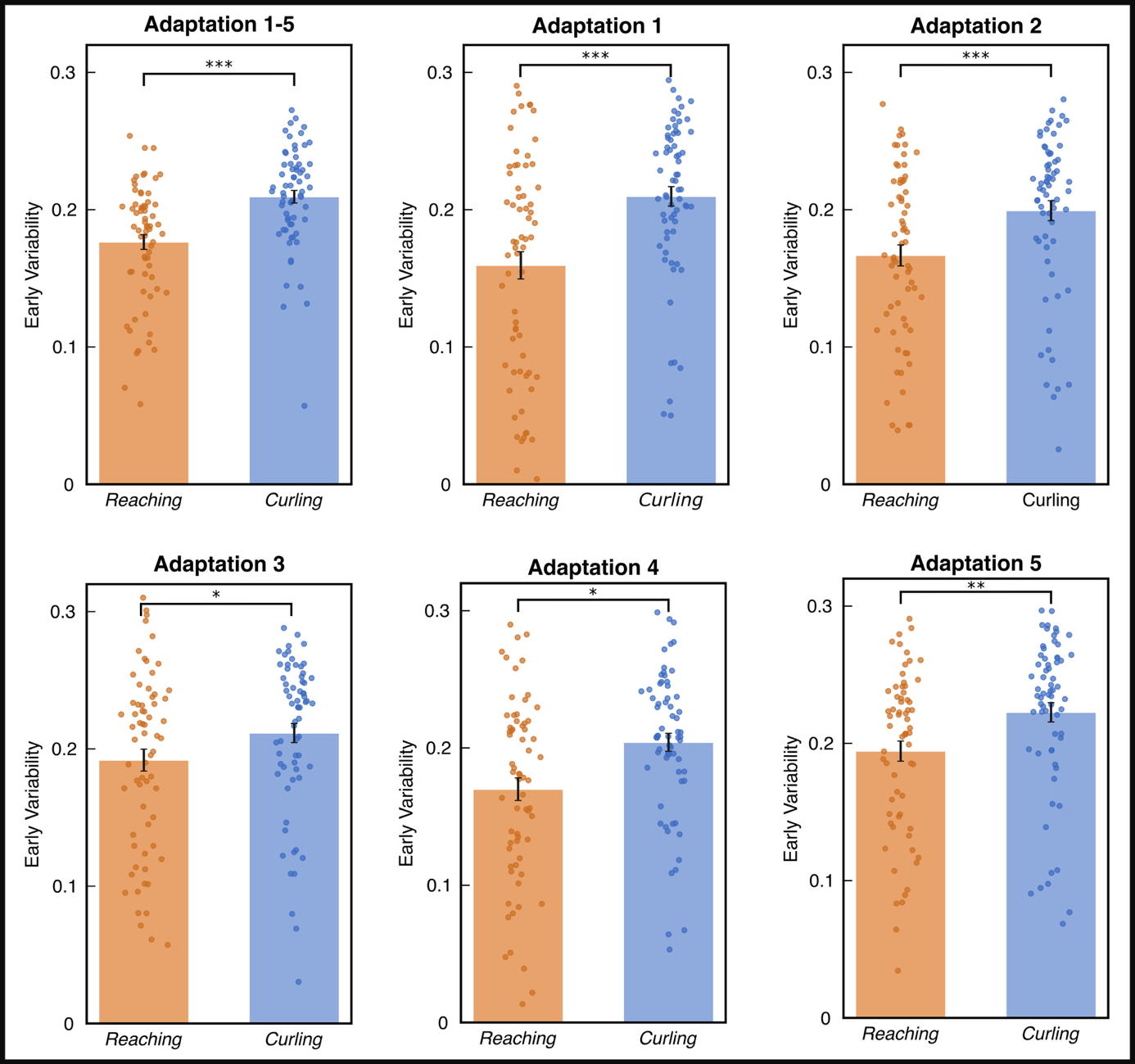
**S3:** **Early** **Motor Variability in the *Reaching* and *Curling* tasks.**

Early motor variability, measured by the standard deviation of the initial five trials, was consistently higher in the *Curling* task compared to the *Reaching* task. This difference was significant for each adaptation block and across all blocks (* *p* < 0.05, ** *p* < 0.01, *** *p* < 0.001).


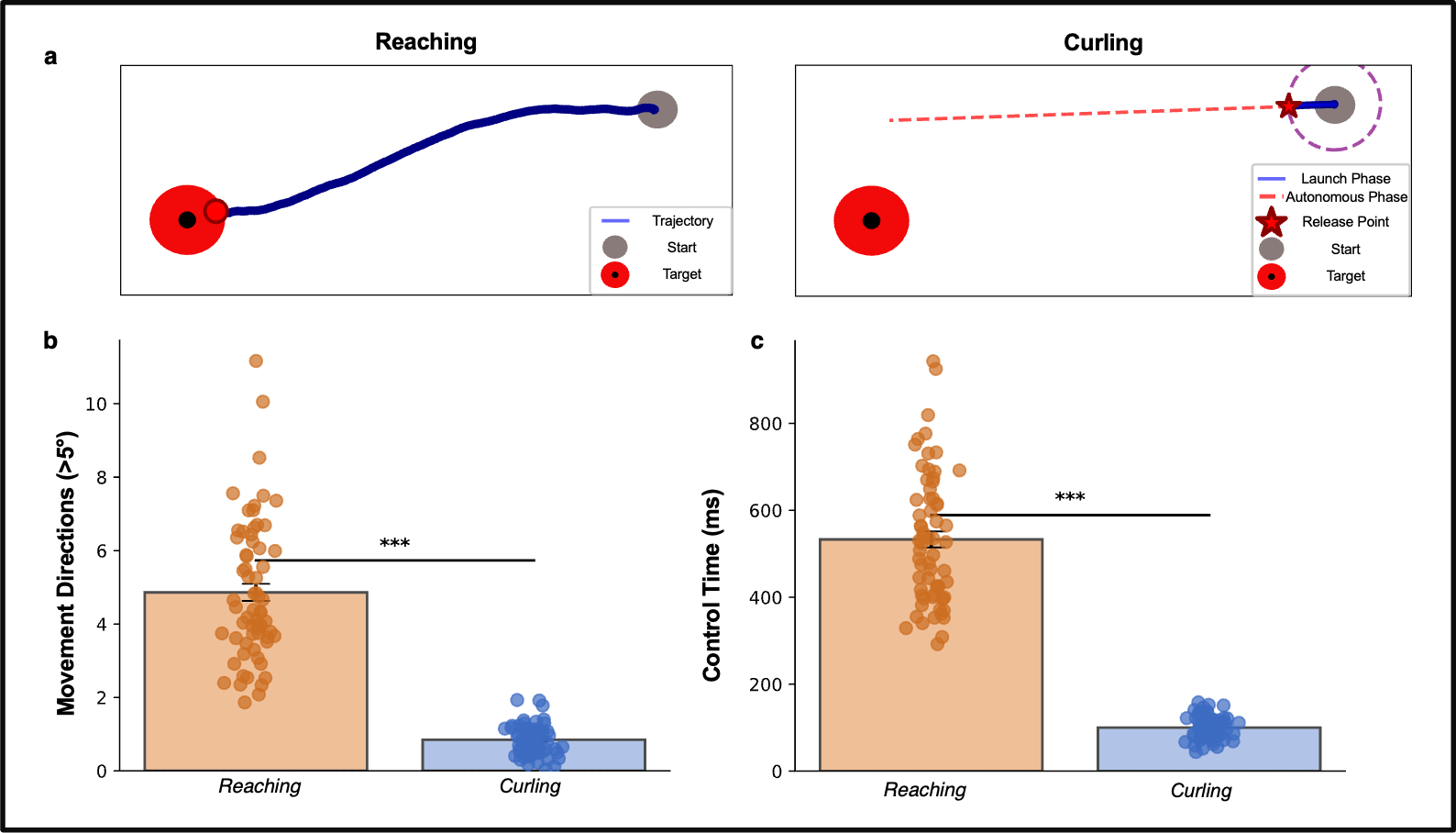

**S4: Movement Trajectory Analyses.**

**a** Representative examples of movement trajectories of a single trial from a single subject for *Reaching* (*left*) and *Curling* (*right*). In *Reaching*, the cursor remained under continuous control (*blue trajectory*) from the start to the target, allowing for online corrections of the ongoing movement throughout each trial. In *Curling*, during the initial “launch phase” the cursor followed the joystick (*blue trajectory*) until it reached the “release point” (*red star*), after which it autonomously continued moving along a straight path (*dashed line*) towards the target (“autonomous phase”). **b** A significantly higher number of distinct movement directions was observed during *Reaching* compared to *Curling* (t_(65)_ = 17.45, *p* < 0.001)*,* which yielded one movement direction on average*.* **c** Control time indicating the time during which subjects were able to perform online corrections of ongoing movements. The control time was substantially longer in *Reaching* compared to *Curling* (t_(65)_ = 23.90; *p* < 0.001). Of note, the very short control time in *Curling* (100.2ms +/- SD 26.25ms) rendered within-trial adjustments of ongoing movements highly unlikely. These results emphasize that *Curling* exclusively relied on *between-trial* learning, while improvements in *Reaching* could arise from both *within-* and *between-trial* learning. (*** *p* < 0.001)

*
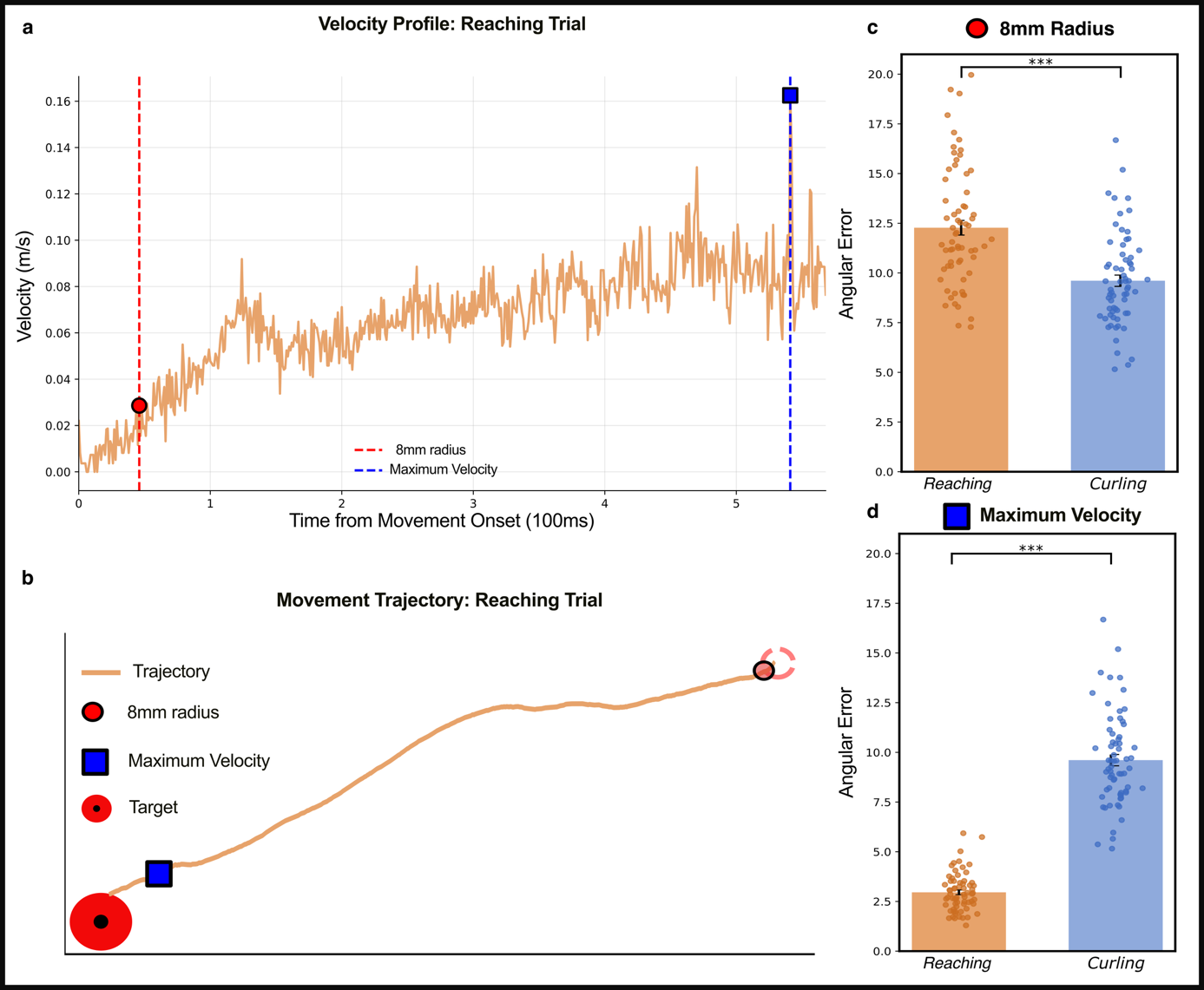
* **S5: Within-trial online correction of movement trajectories during *Reaching*.**

**a** Velocity profile of an exemplary, representative *Reaching* trial of a single subject. The time axis is aligned to movement onset. The red dashed line indicates the time point at which the cursor crossed the 8mm radius (identical to the “release point” in *Curling*). The blue dashed line marks the time point of maximum velocity which was used to calculate angular errors in the *Reaching* task. **b** Movement trajectory of the same *Reaching* trial. The red circle represents the 8mm radius “release point” at which angular errors were measured in the *Curling* task. The red dot marks the cursor position at this release point, the blue square indicates the cursor position at maximum velocity when angular errors were quantified for *Reaching*. **c** At the “release point”, *Reaching* showed significantly higher angular errors than *Curling* (mean ± SEM). **d** Conversely, at the maximum velocity measurement point, *Reaching* yielded significantly lower angular errors than *Curling*, demonstrating that substantial online corrections occurred between the release point and the reference point at peak velocity. Hence, improvements in performance during the *Reaching* task heavily relied on online corrections within ongoing trials. (*** *p* < 0.001)
